# The failure of adherence of the antiretroviral therapy is a field of work for the psychologist to HIV possitive patients in intensive care units

**DOI:** 10.1192/j.eurpsy.2021.1328

**Published:** 2021-08-13

**Authors:** C. Vasilescu, M. Largu, G. Lacatusu, C. Dorobat, C. Manciuc

**Affiliations:** 1 Infectious Diseases, Hospital of Infectious Diseases Iasi, Iasi, Romania; 2 Psychology, University of Medicine and Pharmacy “Gr. T. Popa” Iasi, Iasi, Romania; 3 Infectious Diseases, University of Medicine and Pharmacy “Gr. T. Popa” Iasi, Iasi, Romania

**Keywords:** intensive care unit, antiretroviral therapy, PSYCHOLOGICAL PROFILE, HIV/AIDS

## Abstract

**Introduction:**

HIV infection is currently considered a worldwide pandemic.

**Objectives:**

The objective of this paper is to outline the profile of HIV – positive patients in intensive care units, regarding the psycho-emotional and viral parameters.

**Methods:**

We realized a retrospective study for a period of 36 months, evaluating HIV positive patients in intensive care unit in the “Sf. Parascheva” Infectious Diseases Clincal Hospital Iasi, Romania.

**Results:**

From 1^st^ of Janury 2011 to 31^st^ of December 2013, the HIV/AIDS Regional Centre of Iasi recorded 2649 hospitalizations, of which 18 cases required intensive medical care, 10 males and 8 females. The number of days of hospital admission varied between 4.5 and 32 days in the Intensive Care Unit. Initialy the psychological interview was conducted for 16 of the 18^th^ patients, 2 cases were with severely deteriorated health status that didn’t allowed communication. From them, 7 survived and they were evaluated at discharge from IIntensive Care Unit and also monitored long term, that revealed an increase in adherence to Antiretroviral Therapy and a change in lifestyle.

**Conclusions:**

HIV positive patients that requires intensive care showed a marked immunological collapse due to abandonment of the therapy or late detection. In order to fully accomplish the needs of the HIV positive patient, the infectious diseases specialist must collaborate with the psychologist.

